# Correction: Seroprevalence of diphtheria and tetanus antibodies among children and adolescents in high- and low-immunization coverage areas in the Lao People’s Democratic Republic

**DOI:** 10.1371/journal.pone.0344304

**Published:** 2026-03-04

**Authors:** Yuta Yokobori, Masaaki Iwaki, Miyuki Kimura, Noriko Kitamura, Haru Angelique Hoshino, Bandith Soumphonphakdy, Chansay Patthammavong, Bangone Tannavong, Amphai Khamsing, Phonethipsavanh Nouanthong, Mathida Thongseng, Masahiro Sano, Ayako Masu, Yuriko Egami, Eiichi Shimizu, Shinsuke Miyano, Kento Mitani, Sumiyo Okawa, Moe Moe Thandar, Hyun Kim, Masahiro Yutani, Mitsutoshi Senoh, Masahiko Hachiya

In the Funding section, the grant number from the funder National Center for Global Health and Medicine (NCGM) Intramural Research Fund, Japan is listed incorrectly. The correct grant number is: 22A01, 22K902C01, and 24A2006.
