# Supplementary material for: Seroprevalence of diphtheria and tetanus antibodies among children and adolescents in high- and low-immunization coverage areas in the Lao People’s Democratic Republic
Source: PLoS One. 2025 Dec 30;20(12):e0339672. doi: 10.1371/journal.pone.0339672 (PMC12752958; doi:10.1371/journal.pone.0339672)
Supplement: S1 Table — (DOCX) [file pone.0339672.s001.docx]

|  | | 2023 | 2022 | 2021 | 2020 | 2019 | 2018 | 2017 | 2016 | 2015 | 2014 |
| --- | --- | --- | --- | --- | --- | --- | --- | --- | --- | --- | --- |
| Oudomxay | Namoh | 114.2% | 114.8% | 100.4% | 107.8% | 104.9% | 99.1% | 113.2% | 95.0% | 100.0% | 90.0% |
|  | Houn | 106.5% | 100.8% | 62.8% | 90.2% | 91.7% | 93.6% | 100.6% | 100.0% | 95.0% | 90.0% |
| Xaisomboun | Anouvong | 83.6% | 24.0% | 31.7% | 35.5% | 35.8% | 48.2% | 25.6% | 69.0% | 78.0% | 52.0% |
|  | Longxan | 63.9% | 72.2% | 64.5% | 63.6% | 56.5% | 64.0% | 68.4% | 51.0% | 78.0% | 44.0% |
| Data source: National Immunization Program in MoH, Lao PDR | | | | | | | | | | | |

S1 Table: The trend of Penta3 vaccination coverage over the past 10 years in each target district.
