# Supplementary material for: Seroprevalence of diphtheria and tetanus antibodies among children and adolescents in high- and low-immunization coverage areas in the Lao People’s Democratic Republic
Source: PLoS One. 2025 Dec 30;20(12):e0339672. doi: 10.1371/journal.pone.0339672 (PMC12752958; doi:10.1371/journal.pone.0339672)
Supplement: S2 Table — (DOCX) [file pone.0339672.s002.docx]

S2 Table: Vaccination card possession and percent agreement in vaccination history between card records and recall.

|  | Total (n) | Vaccination card holders (n,%) | Vaccine history on card | Vaccinated by recall | Unvaccinated by recall | Agreement rate (%) |
| --- | --- | --- | --- | --- | --- | --- |
| Overall | 960 | 156,16.3% | Vaccinated | 142 | 8 | 92.3% |
|  |  |  | Unvaccinated | 4 | 2 |  |
| Province | | | | | | |
| Xaisomboun | 480 | 45, 9.4% | Vaccinated | 36 | 5 | 82.2% |
|  |  |  | Unvaccinated | 3 | 1 |  |
| Oudomxai | 480 | 111, 23.1% | Vaccinated | 106 | 3 | 96.4% |
|  |  |  | Unvaccinated | 1 | 1 |  |
| Age | | | | | | |
| 1-4y | 240 | 110, 45.8% | Vaccinated | 101 | 6 | 91.8% |
|  |  |  | Unvaccinated | 3 | 0 |  |
| 5-9y | 240 | 33, 13.8% | Vaccinated | 30 | 2 | 90.9% |
|  |  |  | Unvaccinated | 1 | 0 |  |
| 10-14y | 240 | 11, 4.6% | Vaccinated | 9 | 0 | 100.0% |
|  |  |  | Unvaccinated | 0 | 2 |  |
| 15-19y | 240 | 2, 0.8% | Vaccinated | 2 | 0 | 100.0% |
|  |  |  | Unvaccinated | 0 | 0 |  |
